# Coping strategies adopted by medical residents in dealing with work-related stress: a mixed-methods study

**DOI:** 10.1186/s12909-022-03520-6

**Published:** 2022-06-09

**Authors:** Shamaila Manzoor, Madiha Sajjad, Idrees Anwar, Aisha Rafi

**Affiliations:** 1 Azad Jammu & Kashmir Medical College, University of Health Sciences (Lahore), Muzaffarabad, Pakistan; 2 Islamic International Medical College, RIPHAH International University, Islamabad, Pakistan; 3 Rawalpindi Medical University, Rawalpindi, Pakistan; 4 Shifa Medical College, Shifa Tameer-e Millat University, Islamabad, Pakistan

**Keywords:** Coping strategy, Fatigue, Medical residents, Long working hours

## Abstract

**Background:**

This study was carried out to identify and explore the difference in coping strategies for dealing with stress; adopted by medical residents in surgical and nonsurgical specialties.

**Methods:**

A mixed methods approach, was chosen for this study. In phase one (quantitative); data were collected by a web-based survey, using a structured questionnaire. This survey was carried out in five major teaching hospitals of Rawalpindi & Muzaffarabad in 2020. Purposive sampling was done and data were analysed using SPSS, recording frequencies and major trends. In phase two (qualitative); six focus group discussions were carried out with 24 participants, using semi-structured questions and prompts. Convenient sampling was done from the cohort of phase 1. These focus group discussions, explored the various coping strategies adopted by medical residents. Later, the data were analysed manually for development of codes, sub-themes and themes.

**Results:**

Out of 250 (100%) participants, 146 (58%) participants responded to the online survey. Surgical residents had a higher response rate (60%, *n* = 87) than nonsurgical residents. Moreover, female participation (54%, *n* = 79) was greater than male participation (46%, *n* = 67). The Mann-Whitney U test was statistically significant only for seeking medical advice to cope with stress (*P* = .029). There was no statistically significant difference found between the coping strategies, employed by medical residents.

In focus group discussions, female involvement was more (58% *n* = 14) than their counterparts. Four main themes were developed after data analysis: self-regulation, tailor-made strategies, educational focus and support system. Finally, minor differences were obtained qualitatively; like, socializing is preferred by surgical residents whereas, spiritualism is chosen by nonsurgical residents.

**Conclusion:**

Quantitatively, no significant differences were found between the coping strategies of medical residents, against work-related stress. However, minor differences were obtained qualitatively due to difference in job demands and level of burnout between these two specialities.

## Introduction

Resident training is an arduous period marked with psychological and intellectual changes in resident doctors; while healing ailing humanity. Prolonged duty hours in training are exhaustive, leading to the weariness of doctors [1]. It causes sleepiness, which leads to slowing of sensorimotor and cognitive functions in resident doctors, becoming a safety hazard for both patients and doctors [2]. Exhaustive duty has been strongly associated with fatigue, sleep deprivation and workplace difficulties. Such factors lead to diminished critical thinking and decision making, instigating more medical errors at this professional stage [3, 4].

A literature review has revealed that physicians use various coping mechanisms, such as acceptance, mindfulness, spirituality, socializing, provocative and leisure activities, mainly based on Lazarus coping theory [5, 6]. Frequently, emotion and problem-focused coping strategies are found to be the established habits of doctors. They sacrifice their personal desires over professional demands. However, when there is an imbalance between job demands and one’s capacity to cope, the situation becomes traumatic [7]. Their self-esteem suffers, so; they react by exhibiting anger towards the patients and bitterness to the superiors, with further harm to themselves and to their patients [8]. Commonly, these individuals are more prone to high emotional exhaustion and depersonalization [9] [10]. However, adapting customized coping mechanisms such as binging/eating at night shifts helps to stay alert and awake [11]. Similarly, taking interval from job, leisure activities, switching off intellectually and emotionally from work during off job time, has positive benefit for workers [12]. This strategy is more prevalent in conscientious personalities, mainly female physicians. Subsequently, medical residents use various coping strategies; to reduce their emotional and physical stress created by their high-acuity jobs. Unfortunately, no formal teaching or training has been provided to overcome such job-related issues, and no formal curricular plans are in place to cope with these prolonged duty-related challenges [13, 14].

Therefore, this study was conducted to identify the effects, their associated coping mechanisms and explore the difference between various coping mechanisms used by medical residents. This would help in reviewing and revising the training programs regarding working hours and job-related stress, which in turn would result in better patient care. Therefore, research questions of this study were:

- ‘What are the effects of long duty hours and various coping mechanisms adopted by medical residents of surgical and nonsurgical specialties?’
- ‘What are the differences in coping strategies adopted by the residents of surgical and nonsurgical specialties to deal with stress resulting from long working hours?’

## Material & Methods

A mixed methods study; with an explanatory sequential approach; was carried out from March 2020 to Sep 2020 [15]. A mixed methods study design was considered the best approach to answer our research question. As, quantitative part (phase 1) would identify the effects of long duty hours and various coping mechanisms adopted by medical residents of surgical and nonsurgical specialties (Providing the holistic picture of the research problem). Subsequently, qualitative part (phase 2) would help in exploring and differentiating various coping strategies, on the basis of quantitative results, adopted by the residents of surgical and nonsurgical specialties to deal with stress resulting from long working hours (providing a detailed picture of the research problem).

**Fig. 1:**
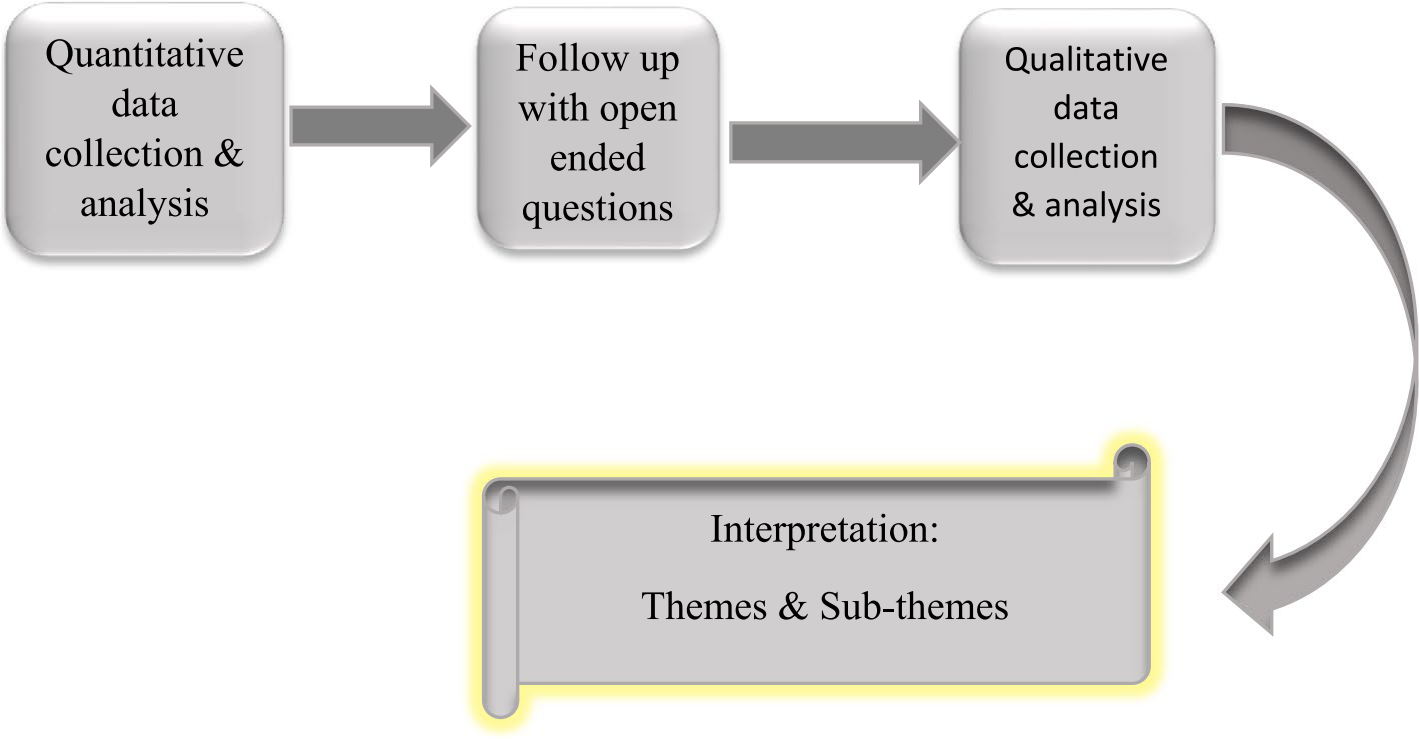
Explanatory Sequential Design (Mixed Methods Study)

The explanatory Sequential design was comprised of two distinct phases where quantitative and qualitative data were collected sequentially (Fig. 1). First, quantitative data were collected followed by qualitative data to explain the quantitative statistics.

The study was conducted after institutional review committee (IRC) approval from RIPHAH International University, Islamabad (Appl# Riphah/IRC/20/123). Subsequently, the study was conducted five major teaching hospitals of Rawalpindi and Muzaffarabad. These teaching facilities are associated with three main universities of Pakistan, two from public sector and one from private sector. Two setting were located in Azad Jammu & Kashmir [Sheikh Khalifah bin Zaid hospital & Abbas Institute of Medical Sciences, Muzaffarabad (affiliated with University of Health Sciences, Lahore)], three were in Punjab, [Holy Family Hospital, Benazir Bhutto Hospital (affiliated with Rawalpindi Medical University) & Pakistan Railway Hospital (RIH affiliated with RIPHAH international University), Rawalpindi]. All the five teaching hospitals are tertiary care level hospitals, with provision of emergency services, ICU, NICU, medicine, surgery, Eye, ENT, Pediatrics, Gynecology & obstetrics. They have state of art emergency medical services, multiple specialties and super-specialties, dedicated senior consultants and a large force of variety of resident doctors having busy schedule, which make all of them an appropriate setting for this study.

Only those medical residents (FCPS year 2, 3 & 4 trainees); from surgical (General Surgery, Gynecology/ Obstetrics & Anesthesia) and nonsurgical (General Medicine, Pediatrics & Radiology) specialties were included in study, who were attending long hour calls (> 80 hrs a week) in surgical and nonsurgical specialties. Off-service residents were excluded from the study as they were not performing extended calls. The sampling technique was purposive, to focus on the certain characteristics (effects & counter mechanism of extended duties) of the study population (medical residents of busy specialties) [16]. So, this sampling provided relevant and rich information, related to the phenomenon of interest. A complete information was provided to all the participants along with informed consent form. Anonymity and confidentiality of data was maintained at all the levels of research.

Therefore, in phase 1, a structured, online survey questionnaire was developed by researchers (educationist from three renowned medical universities) after a thorough literature review; following the PRISMA-P protocol, and following seven steps of research tool development. Questionnaire reliability was assessed by test-retest, inter-rater and split half methods. Likewise, face validity of questionnaire was conferred by medical educationists and relevant subject experts. Subsequently, its piloting was done with a small sample to check the feasibility of the survey. The reliability of the online questionnaire has been assessed using Cronbach’s Alpha. Since the value of the Cronbach’s Alpha for all 24 items in the questionnaire is 0.766, which is more than 0.70. So, the items in the questionnaire are consistent and reliable.

**Fig. 2:**
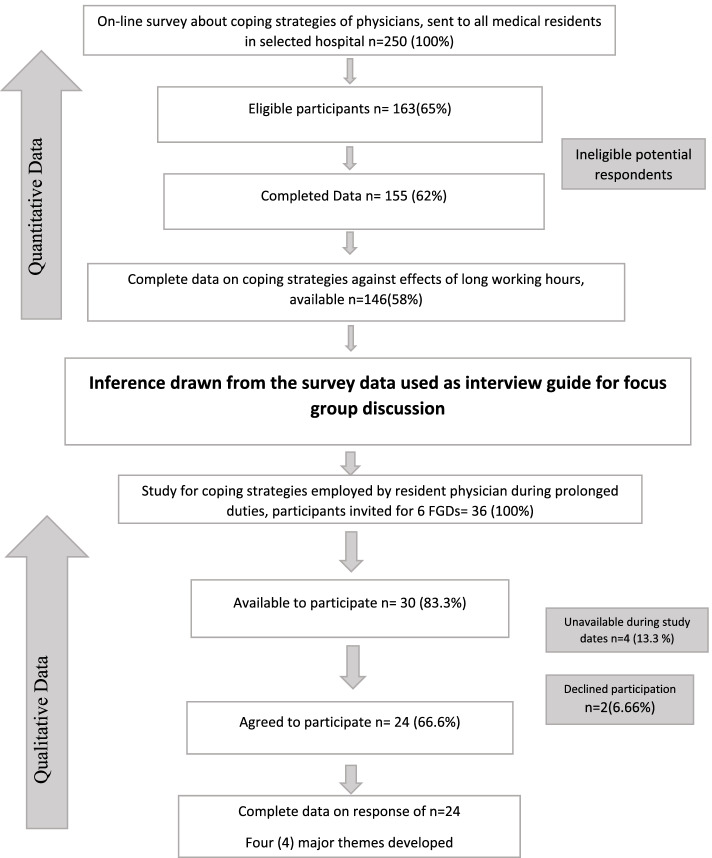
Mixed Methods study Design Flow chart

Later, this questionnaire was circulated among the maximum number of participants (*n* = 250) more than the sample size calculated. This was keeping in view the possibility of the dropouts or failure to respond. A total of 146 PGTs participated in this online survey. Therefore, the response rate was 58% (*n* = 146) out of 250 participants. Statistical analysis of quantitative data was done on statistical package for the social sciences (SPSS version 22). Data was entered in SPSS, a nonparametric Mann- Whitney-U test was applied to the data to find a statistically significant difference in the coping strategies adopted by surgical and nonsurgical medical residents. Compilation, analysis and interpretation of quantitative data identified specific trends in the coping strategies of medical residents. On the basis of quantitative results a semi structured questionnaire was developed for FGDs. Qualitative part, was based on phenomenology approach which helped to understand the nature of phenomenon of long working hours; resident’s perception of this phenomenon and countermeasures used by medical residents, performing long duties. Subsequently, analysis and comparison of these countermeasures has provided a detailed picture of the research problem. Therefore, six focus group discussions (FGDs) were conducted involving 24medical residents, until data became saturated. Duration of each FGD was 60–90 minutes. These FGDs were based on semi-structured questions and prompts developed after analysis of quantitative data by researchers and given to participants during FGDs. Convenient sampling was done from the cohort of phase one. In phase II, qualitative data were collected; after these FGDs, data were transcribed verbatim. Data handling was done manually, and open and axial codes were derived from the data. Thematic analysis of qualitative data resulted in subthemes and theme formation (Fig. 2). The rigor of the study was maintained by convenient sampling from cohort of phase I, good sample size, member checking and proficient data analysis.

## Results

Out of 100% (*n* = 146) of the participants; 54% (*n* = 79) were females, while 46% (*n* = 67) were males. The average age of the participants was 27_ + 1 year. Surgical disciplines had a response rate of 60% (*n* = 87), and nonsurgical disciplines had a response rate of 40% (*n* = 59). Among them, 82 % (82%, *n* = 120) of participants worked for more than 80 hours a week. The variables studied (effects of long working hours) were exhaustion (67.12%, *n* = 98), lost from surroundings (58.90%, *n* = 86), errors or negligence at work (26.06%, *n* = 38), disturbed relations with the family (63.01%, *n* = 92) effect on academic performance (59.59%, *n* = 87) and skipping meals (85.62%, *n* = 125) (Table 1).

**Table 1:** Statistical Analysis of the effects of prolonged duties

Sr. no	Survey questions about effects	Frequency (n)		Percentage (%)	Value Man-Whitney Test	
		Occasionally/Rarely	Frequently/ very frequently		Value	***P***-Value
**1**	Feeling sleeping at work	87	57	39.04%	2388.00	.441
**2**	Slowing of reflexes	70	74	50.68%	2512.00	.962
**3**	Tiredness at work (during extended duties)	47	98	67.12%	2415.00	.641
**4**	Lost from surroundings	59	86	58.90%	2206.00	.177
**5**	Chances of error at work place	106	38	26.02%	2393.50	.573
**6**	Detachment from environment	72	69	47.26%	2559.00	.975
**7**	Strained relation with family	48	92	63.01%	2478.50	.712
**8**	Irregular/skipping meals	21	125	85.62%	2222.00	.131
**9**	Effects on physical & mental health	84	61	41.78%	2326.00	.312
**10**	Effect on educational performance	54	87	59.59%	2562.00	.985
**11**	Effect of educational failure on performance at work	60	83	56.85%	2243.00	.549

The coping mechanisms were taking caffeinated drinks (72.38%, *n* = 107), talking to their colleagues (43.15%, *n* = 63) and eating/munching (55.48%, *n* = 81). Nevertheless, the majority of doctors neither smoked (91.7%, *n* = 134) nor used drugs/alcohol as coping mechanisms (95%, *n* = 139); likewise, error in the workplace was occasional (Table 2).

**Table 2:** Statistical Analysis of web-based surveys about coping strategies

Sr. no	Survey Questions about coping	Frequency (n)		Percentage (%)	Value Man-Whitney Test	
		Occasionally/Rarely	Frequently/ very frequently		Value	***P***-Value
**1**	Formal training/ stress management workshop at start of training	140	6	4.11%	2524	0.626
**2**	Frequency of power naps to remain alert	133	13	8.90%	2536	0.895
**3**	Frequency of micro breaks to remain fresh	113	33	22.60%	2268	0.201
**4**	Intake of caffeinated drinks	39	107	73.28%	2333.5	0.311
**5**	Help from seniors/mentors to cope with long duties	109	37	25.34%	2109.5	0.058
**6**	Seeking social support as coping mechanism	98	48	32.87%	2372	0.42
**7**	Talking to friends and family	99	46	31.51%	2420	0.541
**8**	Talking to colleagues as coping strategy	81	63	43.15%	2349	0.461
**9**	Food as mood elevator	65	81	55.48%	2481	0.723
**10**	Seeking medical advice	98	48	32.88%	2044	0.029
**11**	Drug/Alcohol intake as coping strategy	126	7	4.79%	2500	0.657
**12**	Smoking at work place	133	12	8.22%	2507	0.74
**13**	Any incentive at work	139	7	4.79%	2481	0.357

^a^5__very frequently, 4__ frequently, 3____ occasionally, 2____ rarely, 1___ Never

Then, the Mann-Whitney U (MWU) test was applied, and the resultant *P*-values were statistically nonsignificant (*P* > 0.05) in all cases, with one exception, that is: seeking medical advice to cope with the deleterious effects of long duties (*P* = .029). Hence, there was no statistically significant difference found between the coping strategies, employed by medical residents.

The reliability of the online questionnaire has been assessed using Cronbach’s Alpha. Since the value of the Cronbach’s Alpha for all 24 items in the questionnaire is 0.766, which is more than 0.70. So, the items in the questionnaire are consistent and reliable.

### Qualitative results

A total of six FGDs were carried out, with twenty-four [17] residents. In these FGDs, the female response rate was higher (*n* = 14, 58%) than that in males (*n* = 10, 42%). After data analysis, four main themes were developed; self-regulation, tailor-made strategies, educational focus and support system (Table 3).

**Table 3:** Qualitative study Findings

Themes	Sub- themes	Axial coding	Open coding
Self- regulation		Time management	Extended duties, compromised self- care and sleep
		Self-control	Reflection on actions, controlling mood swings, Requesting rotational duties
		Countering emotions	Self-counselling, seeking senior help, fed-up from duty
Tailor made strategies	Dietary	Caffeinated drinks	Tea/coffee resulted in improved decision making, better critical thinking and increase alertness
		Eating/Binging	Munching freshens, lessen effects of fatigue
		Smoking/Alcohol	Nicotine, stimulant
	Self- survival	Micro naps	Small sleep spells controls altered circadian rhythm; headaches due to sleeplessness
		Water splashes/ablution	water splashes maintain alertness/freshness, washes for praying to relive anxiety and anger
		Praying	Spiritualism cope negative emotion, enhances positivity
		Micro breaks	Little breaks and small pauses, relaxation
		Social media usage	Using internet, face book keeps up-to-date and alert
		Controlling digital clock	Shrinkage of personal time, reducing sleep time and self-care
		Walking	Physical exercise maintains alertness and relieve mental exhaustion
Educational Focus	Prioritizing studies	Sufferings due to studies	Compromise on sleep time, personal time, family and friend time, leisure time
		Policy makers concentrate	Increase human resource (doctors), reduction in working hours, MOCK exams
Support System	Consulting seniors and Peers	Seeking help from colleagues	Seeking seniors’ help, taking little breaks, talking to staff around
	Support of friends and family	Family matters	Hanging out with friends, obeying spouses quietly, doubling duties so can take day off, talking to friends and family, leisure time with family

### Self-regulation

The mental, physical and psychological status along with circadian rhythm was altered due to frequent, long working hours. Most medical residents adopt the regulation of time to handle deleterious effects efficiently. So, they pre-planned and handled their concerns tactfully.

… … ..’ I have to manage several things before coming to a long duty’ … ...

Similarly, reflection on their own actions and self-control was another approach that produced a win-win situation.

… ….’ I cope by self-counselling, that this spell of hardship is for 4 years, good times will come’ ……

### Tailor made strategies

The adaptation of coping strategies by medical residents is found according to their requirements/preferences. The majority of residents practice frequent caffeine intake, eating/binging, water splashes on the face and ablution to avoid sleepiness, especially; during night calls.

… ...’I overcome to work related fatigue by drinking tea, more caffeinated drinks make me fresh’ … ...

Some seek refuge in praying. However, some believe in taking micro-breaks (breaks for 10—15 min) and micro naps (sleep for 10----15 mins) to combat tiredness and sleepiness. Boys prefer smoking, whereas; girls use social media to freshen up during long exhaustive duties.

… … ‘to overcome sleepiness I use water splashes, breathe in fresh air, using phone and internet facilities’ ……

These tactics maintain alertness, decision making and critical thinking of the residents on call. Using humour, joking with staff and laughing about the stressful incidences were also the coping strategies used by the residents.

### Educational focus

Prolonged duty hours have grave consequences on the educational activities of these residents. They usually miss teaching rounds/morning classes and elective operation lists; due to late night calls. Furthermore, reported by many that they are physically present in class but mentally absent; therefore, do not learn much. Nevertheless, medical residents counter these difficulties by reducing their sleep time (spending more time on studies), reducing time for self-care, spending less time with friends and family and shrinking leisure time.

… ...’I prepare my assignments and presentations at the expense of my personal and family time’ … ...

… … ‘for passing exams we have to study a lot, which is affected by prolonged duties, so I have to compromise my sleep for preparing me next day round and quiz’ … ...

### Support system

The support system encourages one to swim swiftly through the tiresome ocean of training. This support system comprises family and friends, colleagues, peers and staff on duty.

… … ‘I cope by spending time with family so I get relax and become stable’ …… .

Similarly, seeking advice from seniors/colleagues for patient safety was frequently stated by many.

… ..’ I ask help from my colleagues to double check my findings & I reflect upon, whether I have missed something’ … … .

This support system has been identified as an important pillar of professional support. Seniors and colleagues definitely helped their juniors; in times of need (exhaustion, poor alertness or sleepiness). Similarly, leisure time with family and hanging out with friend kept the boat rowing. Moreover, debriefing by the consultant after a challenging case; provided unconditional emotional and educational support to the residents, as stated by participants.

Negative emotions are not very common among residents, these emotions usually arise as a result of thankless behavior & abusive language of patient’s attendants, though rarely from patient, himself. Which result in to regret and rage in residents towards carrier selection, therefore, impeding smooth execution of health provision to patients. However, these problems are generally solved by the guidance of senior members of the team.

… … ‘when people misbehave, despite all of my efforts for pt. then I think that it’s better to select another profession. Such negative emotions are coped by self- counselling and by consulting friends & senior colleagues which satisfies me quite well’ ……

## Discussion

The current study has identified and explored; the difference in coping strategies of medical resident, in dealing with work related stress. This complex phenomenon of coping, depends on several factors, like, personality type and work demand along with long working hours. In the quantitative phase of study, no differences in effects or coping strategies were found, of both specialities. However, qualitative phase, explored the problem by using semi-structured interviews, based on findings of quantitative phase [18]. Subsequently, minor differences have been reported between coping mechanism of surgical and nonsurgical specialities, residents.

This study has shown that some coping strategies were preferred by surgical residents, like, socializing is preferred by surgical residents whereas, spiritualism is chosen by nonsurgical residents. Minor differences among coping mechanisms were probably due to differences in job demands and levels of burnout of residents between the two specialties [19]. Likewise, the tailor-made strategies used by participants of this study were caffeinated drinks, eating & smoking, praying and social media usage to reduce their stress during extended duties. A similar study showed that positive philosophic out-look, spirituality, power naps, micro breaks, exercising and personal sacrifices are used as provocative measures by residents to overcome the effects of long duties [12]. In this study many participants use spiritualism (praying) to cope negative emotions and for self-survival. A comparable study concluded that acceptance, active coping and spiritual attitudes was associated with lower emotional exhaustion and depersonalization (< 0.005) [10]. These coping mechanisms make them alert both physically and mentally, helping them to make efficient decisions for their patients.

A similar study showed that both emotion-focused and problem-focused coping strategies were used by the residents. These coping habits become the established habits of doctors to sacrifice their personal desires over the professional demands [7]. Reflection on actions, controlling mood swings and self-control was found; prominent coping mechanisms, of the medical residents in this study. Similarly, stress management technique, reflections, shared experiences, resilience training has been shown by a comparable study [20]. This self-control would focus their critical thinking and energy on their goal.

The novel coping strategies adopted by the medical residents working in various hospitals in Pakistan revealed by this study are, that participants control negative emotions through self-counselling and social support systems. They seek help from their families, friends, colleagues and other duty staff. Correspondingly, a study showed that physician stress has been addressed by various means, such as meditation, gratitude, exercise and healthy relations with friends and family [14]. Likewise, leisure time with family, taking little breaks from job and workout has been reported as coping tactics by the participants of this study. Whereas, strategic planning for future; taking time out, physical exercise and family time also serve to detract from work related stress [5]. Moreover, a recent study in Australian health-workers have shown that their most commonly reported adaptive coping strategies were maintaining exercise and social connection [21].

Naturally, frequent extended duties result in altered circadian rhythm. This altered rhythm was managed by Pakistani residents either by taking power naps or drinking plenty of caffeinated drinks. Similar, tactics were opted in a former study; by anaesthesia residents, that is, caffeine intake, strategic naps, micro-breaks, controlled exposure to bright or blue enriched light during overnight shifts and appropriate use of post-call recovery sleep [22]. These tactics do not interrupt the patient care yet lessen the deleterious effects of long duty hours.

Furthermore, the fundamental goal of residency training is turning novices into experienced professionals, who would serve humanity as compassionate and competent healers. Therefore, didactic and clinical education is the right of residents, and sufficient time and energy of his or her should be reserved for it. Therefore, educational setbacks are countered by personal compromises on sleep, family and leisure time. These positive strategies resulted in lower emotional exhaustion sand depersonalization. As shown by a study, that less emotional exhaustion and depersonalization is seen in the residents who seek compromise and productively resolve issues [10]. Likewise, debriefing sessions by the consultant after a challenging case; provided unconditional emotional and educational support to the residents, as stated by participants. The literature also cited that debriefing sessions are emotional and social support for attendees [23]. However, academic stress is common among students but majority could not handle it tactfully. Therefore, it should be addressed during counselling sessions [24].

Moreover, support from family and friends has been identified as an important pillar of professional support. Leisure time with family and hanging out with friend kept the boat rowing. Other strategies used frequently by participants were talking to the co-workers and sharing humour at the workplace. Nevertheless, personal compromises (over sleep, tiredness) seemed essential to tactfully handle the differences, especially with loved ones/spouses. Therefore, a collaborative program of cognitive behavioural therapy, mindfulness and coping skills should be adopted; and implemented for residents, as shown by the literature [17]. A similar study has shown that psychological interventions which can reduce perceived stress, strengthen social support and a positive coping style may be helpful to medical doctors to overcome deleterious effects of job related stress [25].

The reliability and authenticity of the study were ensured by maintaining the anonymity and confidentiality of participants and a high response rate. Member checking and triangulation was done. Reflexivity was ensured.

Limitations of the study included; a restricted number of FGDs due to the pandemic (travel restrictions). Therefore, multicentric views of residents could not be gathered. Similarly, health safety limited the mobility of researchers to hospitals.

As, this research has shown that medical residents have formulated their own coping strategies. This study has proposed, that curriculum revision and universal policy making; is need of hour, for future resident’s programs. Moreover, resident’s emotional maturity, challenging coping and humbleness should be incorporated in the selection criteria, in addition, to board scores, references and interviews.

Further studies are required to explore the evolving challenges of prolonged duties; on medical residents in the present scenario of digitalization.

## Conclusion

There was no significant difference found in the coping strategies; used by residents of surgical and nonsurgical specialties. However, minor variation does exist, as surgical people are more prone to both mental and physical exhaustion; than their parallels; due to the nature of their job. Surgical residents are more inclined towards eating and socialization with their dear ones, whereas, nonsurgical residents use spirituality as the mainstay.

## Data Availability

The datasets supporting the conclusions of this manuscript are included within the article (and its additional files).

## Abbreviations

FGD: Focus group discussion
IRC: Institutional review committee
UHS: University of Health Sciences
RMU: Rawalpindi Medical University
RIH: RIPHAH International University
ICME: International conference of medical education
